# Malignant glomus tumor of the index finger

**DOI:** 10.4322/acr.2020.184

**Published:** 2020-09-02

**Authors:** Tarun Kumar, Iffat Jamal, Jitendra Singh Nigam, Jagjit Kumar Pandey

**Affiliations:** a All India Institute of Medical Sciences, Department of Pathology. Patna, Bihar, India.; b All India Institute of Medical Sciences, Department Surgical Oncology. Patna, Bihar, India.

**Keywords:** Neoplasms, Vascular Tissue, Paraganglioma, Extra-Adrenal, Pericytes

## Abstract

Glomus tumor (GT) is a benign mesenchymal tumor with an estimated incidence of 1.5 to 2% of soft tissue tumors. The majority of glomus tumors are benign and are mostly seen in the superficial skin & soft tissue of upper and lower distal extremity. The malignant variant of the glomus tumor is scarce. We report a case of a recurrent glomus tumor diagnosed in a 28-year-old male patient, who complained of painful swelling in the proximal phalanx of the right index finger. The magnetic resonance imaging of the hand revealed a well-defined multilobulated soft tissue mass at the palmar aspect of the 2^nd^ digit along the shaft of the proximal phalanx. Histopathology revealed a well-circumscribed tumor arranged in solid sheets, nests and cords interconnect by vessels of varying size. The tumor cells were round to oval, showed moderate nuclear pleomorphism, eosinophilic cytoplasm, atypical mitoses (>5/10HPF), and necrosis. Immunohistochemically tumor cells reveal diffuse and strong cytoplasmic positivity with smooth muscle actin (SMA). Based on histomorphology and immunohistochemistry, a final diagnosis of malignant glomus tumor was made. We report this case due to its rarity, and it to be included among the differential if the lesion is painful and recurrent.

## CASE REPORT

**Figure 1:**
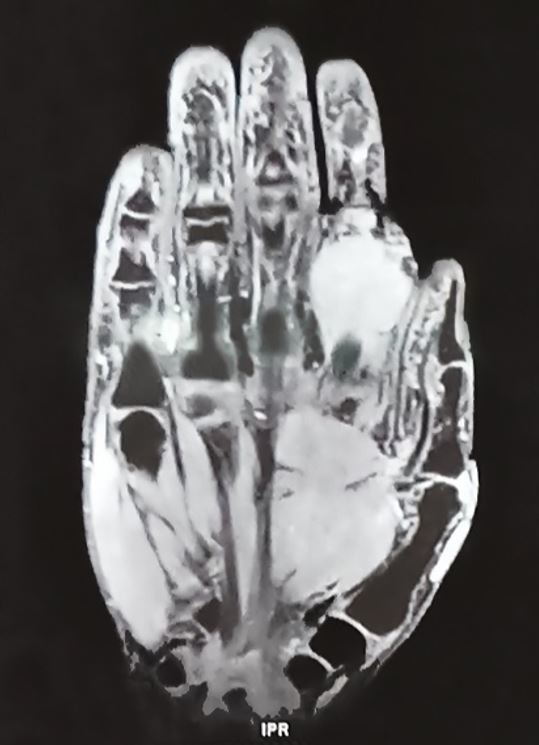
MRI of the right hand, T1 weighted image showing a well-defined multilobulated mass.

A 28-year-old male presented with painful swelling in the flexor aspect of the proximal phalanx of the right index finger for one year. His medical history included a similar lesion at the same site four years before, which was excised, and the histopathological diagnosis was of a glomus tumor. The present swelling was firm, tender with restricted mobility from the underlying structure. The magnetic resonance imaging (MRI) of the hand revealed a well-defined multilobulated soft tissue mass at the palmar aspect of the 2^nd^ digit along the shaft of proximal phalanx originating and encasing the flexor tendon sheath (Figure 1).

**Figure 2:**
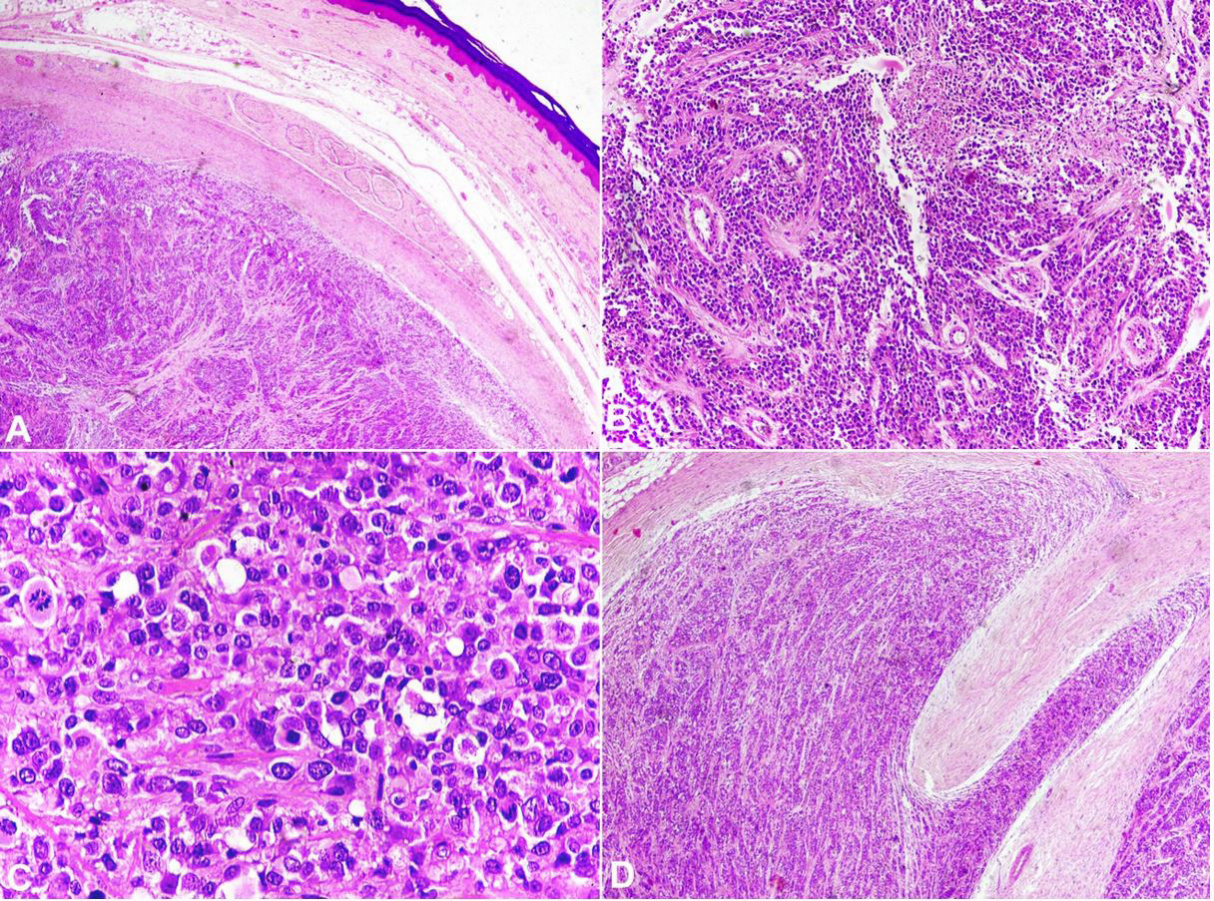
Photomicrographs of the tumor. **A –** shows a well-circumscribed tumor within the dermis arranged in solid sheets and nest (H&E, x2); **B –** the tumor cells are round to oval, show moderate nuclear pleomorphism and eosinophilic cytoplasm. Note the necrosis (H&E, 10x); **C –** few bizarre cells and atypical mitoses seen. (H&E x40); **D –** Tumor cells are Infiltrating the adjacent soft tissue (H&E x4).

**Figure 3:**
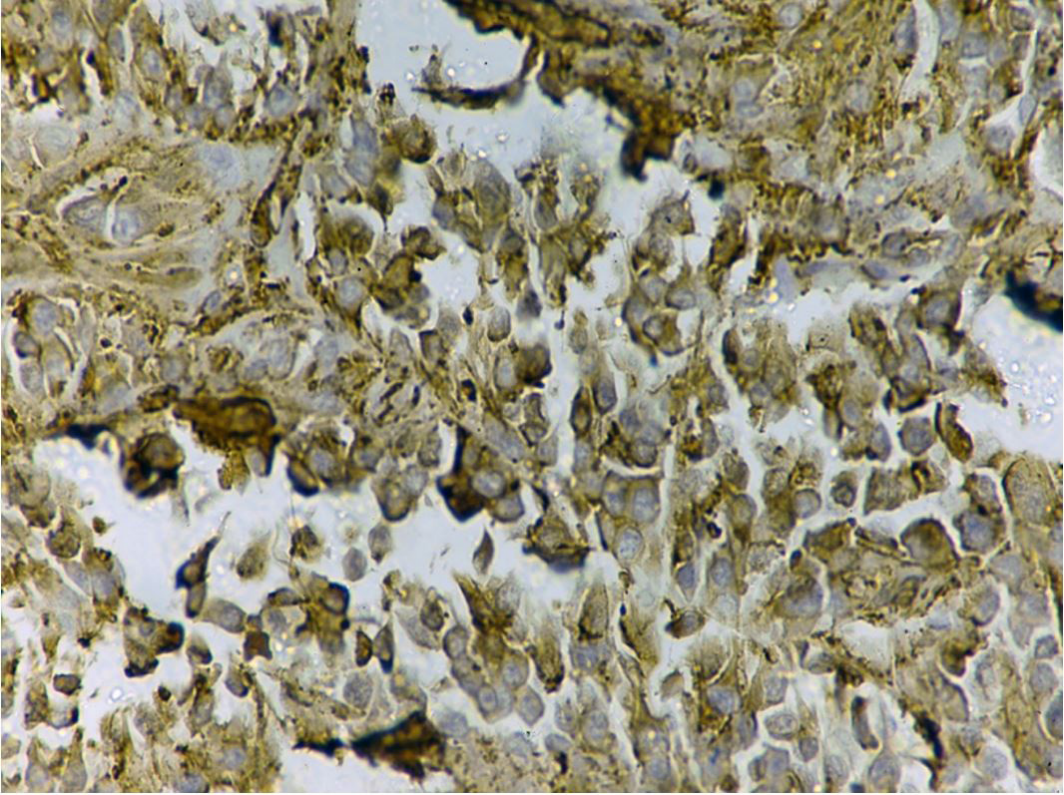
Photomicrograph of the tumor showing a positive reaction for SMA (40X).

Radiological differentials of giant cell tumor of the tendon sheath and fibrous tumor of the flexor tendon sheath were rendered. Surgical amputation of the right index finger was done and sent for histopathological examination. The histopathology revealed a well-circumscribed tumor arranged in solid sheets, nests, and cords interrupted by vessels of varying size and fibrous bands (Figure 2A). The individual tumor cells were round to oval, show moderate nuclear pleomorphism, and a moderate amount of eosinophilic cytoplasm. (Figure 2B) Few bizarre cells, atypical mitoses [>5/10 high power field (hpf)], and necrosis were also noted (Figure 22C). Osteoclast-like giant cells were not seen. The tumor was infiltrating the soft tissue (Figure 2D). However, the underlying bone and overlying skin were free of tumor. Based on radiological and histopathological findings, we considered the following differentials diagnosis. 1) Poorly differentiated carcinoma; 2) Giant cell tumor; 3) Melanoma. Giant cell tumor was ruled out due to the absence of giant cells. Immunohistochemically tumor cells reveal diffuse strong cytoplasmic positivity with smooth muscle actin (SMA) (Figure 3) while negative for cytokeratins, HMB-45, and SOX-10. Based on histomorphology and immunohistochemical findings, a final diagnosis of malignant GT was made. The immediate postoperative period and the 6-month follow-up were uneventful.

## DISCUSSION

GT, also known as Glomangioma, is a distinct, benign, rare mesenchymal tumor with an estimated incidence of 1.5 to 2% of soft tissue tumors.^1^ Glomus tumor mostly occurs in the superficial skin and soft tissues of the upper (such as subungual region, palm, wrist, and forearm) and lower distal extremities. GT can be found in the deep dermis, trachea, lung, mediastinum, upper and lower gastrointestinal tract, urinary bladder, and bone.^1^ ^-^ ^5^ In 1924, Masson^6^ first described that GT arose from perivascular cells of the glomus body, a modified form of arteriovenous anastomosis (the Sucquet-Hoyer canal) that helps in thermal regulation. The majority of the glomus tumor is benign. However, the malignant variant is quite rare. There is no sex predilection; however, subungual located glomus tumors are mostly seen in the female population.^7^ The mean age of diagnosis is 20-40 years. The majority of the cutaneous glomus tumor presents as single, small blue-red nodules with paroxysmal pain, which are either elicited by cold exposure or even by minor tactile stimulation. Multiple GTs are often found in the children and are due to the mutation in the glomulin gene located on chromosome 1p21-22, which shows an autosomal dominant pattern of inheritance.^8^ ^,^ ^9^ Also, an association of solitary and multiple subungual GT with von Recklinghausen disease (neurofibromatosis type 1) has been described.^10^ In 1972, Lumley and Stansfeld^11^ first reported a case of atypical glomus tumors. Atypical GT was classified into a locally infiltrative glomus tumor. In 1990, Gould et al.^12^ reported six cases of locally aggressive and/or potentially malignant GT, and proposed the following classification: (i) locally infiltrative GT, (ii) glomangiosarcoma arising in a benign glomus tumor, and (iii) glomangiosarcoma arising de novo. In 1996, Brathwaite and Poppiti^13^ first described a malignant glomus tumor based on a metastatic clinical feature in a patient with multiple glomus body hamartoma. WHO^14^ classified the glomus tumor based on the tumor location, size, nuclear atypia, atypical mitosis, and mitotic activity into (i) malignant GT; (ii) GT of uncertain malignant potential; (iii) Symplastic GT; (iv) Glomangiomyoma; and (v) Glomangiomatosis. WHO criteria for malignant glomus tumor are marked nuclear atypia, any level of mitotic activity, or atypical mitotic figures.^14^ Folpe et al.^15^ studying 52 cases of GT, described two patterns of malignant glomus tumor namely (i) one of the patterns shows sheets of round cells which architecturally resemble a benign glomus tumor having eosinophilic cytoplasm with centrally placed round or oval nucleus, high nuclear/cytoplasmic ratio, moderate to marked nuclear pleomorphism, typical or atypical mitotic figures, and numerous tiny blood vessels scattered between the tumor cells; and (ii) a less common pattern which is cytoarchitecturally differ from a benign glomus tumor and is composed of the spindle or fusiform cells arranged in short fascicles. Immunohistochemically, tumor cells show positivity for (SMA), and type IV collagen. In our case, the size of the tumor was 2.6 cm in its longest axis, deeply located, presented atypical mitosis, mitotic activity > 5-6/50 hpf, and areas of necrosis. No distant metastasis was present in the index case. The poorly differentiated carcinoma, Giant cell tumor, melanoma were considered as differential diagnoses, and were ruled out based on histomorphology and immunohistochemistry. As the patient did not provide the previous histological material to confirm the nature and diagnosis of the initial tumor, we accepted the initial histopathology report as correct. Hence, the diagnosis of malignant transformation of a glomus tumor was rendered. The recurrence and malignant transformation of glomus tumor are described in literature.^16^ Malignant glomus tumor arising in proximal phalanges is a rare entity. To date, not more than 10 cases are reported in English literature.^17^ ^-^ ^22^

## CONCLUSION

A recurrent benign glomus tumor with malignant transformation is occasional. Such malignant glomus tumors are notorious for recurrences, metastasis, and even death. A careful clinical, radiological, histopathological, and immunohistochemical correlation is a must to reach a correct diagnosis and treatment.
